# Supplementary material for: Keeping up with the genomes: efficient learning of our increasing knowledge of the tree of life
Source: BMC Bioinformatics. 2020 Sep 21;21:412. doi: 10.1186/s12859-020-03744-7 (PMC7507296; doi:10.1186/s12859-020-03744-7)
Supplement: Supplementary file 1 — Additional file 1 The number of taxonomic labels per year. The number of updates in the NCBI bacteria genome database on six taxonomic levels, namely, species, genus, family, order, class and phylum. We have a figure for “Accumulative number of updates per year” per taxonomic level (A, B, C, D, E, F) and a figure for “compared with last year, the number of new updates per year” per taxonomic level (G, H, I, J, K, L). [file 12859_2020_3744_MOESM1_ESM.pdf]

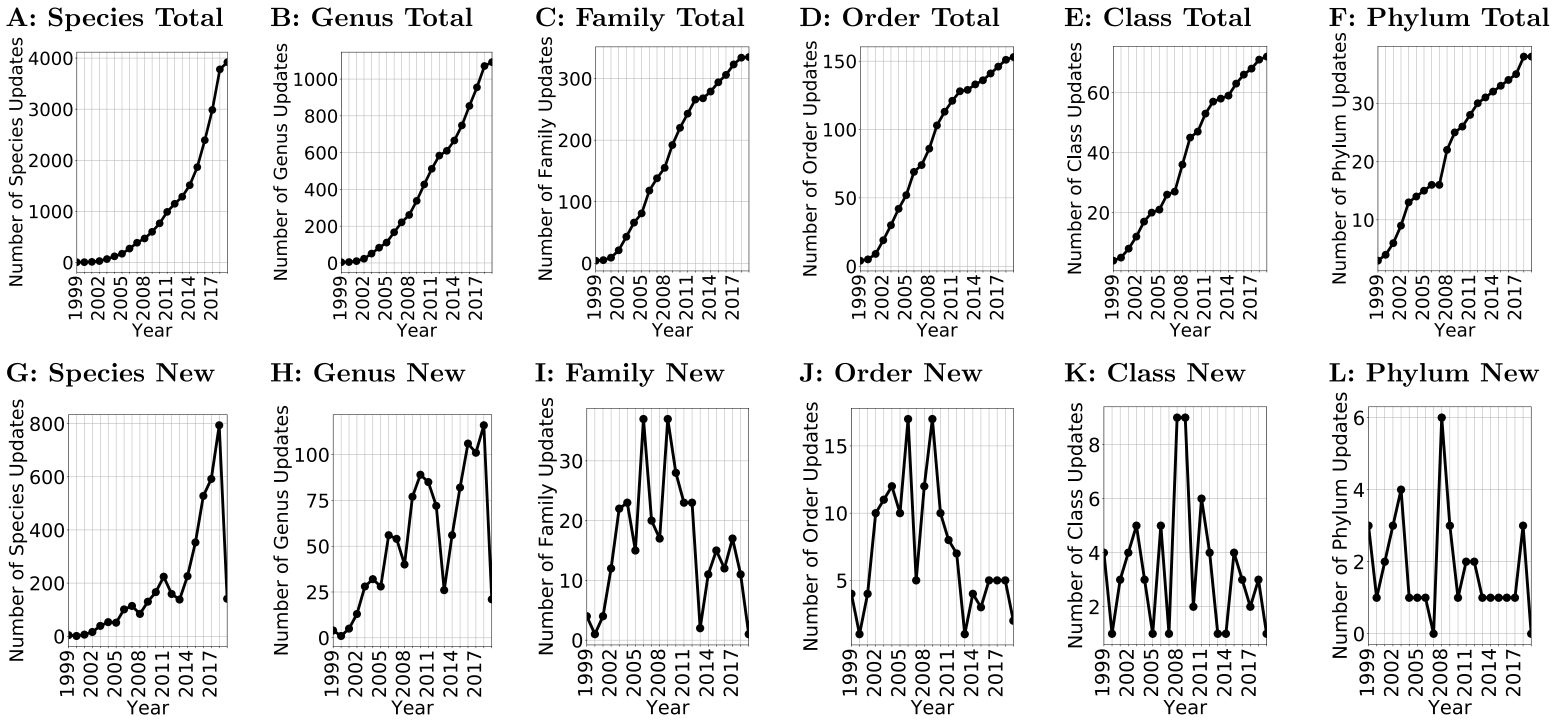
A: Species Total
B: Genus Total
C: Family Total
D: Order Total
E: Class Total
F: Phylum Total
G: Species New
H: Genus New
I: Family New
J: Order New
K: Class New
L: Phylum New
Number of Species Updates
Number of Genus Updates
Number of Family Updates
Number of Order Updates
Number of Class Updates
Number of Phylum Updates
Year
1999
2002
2005
2008
2011
2014
2017
