# Supplementary figures and images for: Keeping up with the genomes: efficient learning of our increasing knowledge of the tree of life
Source: BMC Bioinformatics. 2020 Sep 21;21:412. doi: 10.1186/s12859-020-03744-7 (PMC7507296; doi:10.1186/s12859-020-03744-7)

**A: 1999**

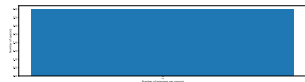

**B: 2000**

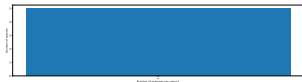

**C: 2001**

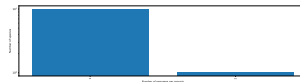

**D: 2002**

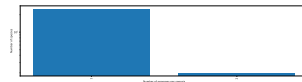

**E: 2003**

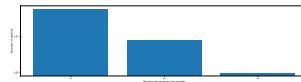

**F: 2004**

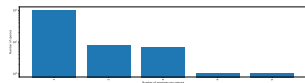

**G: 2005**

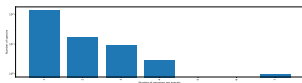

H: 2006

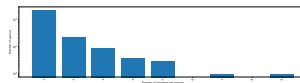

**I: 2007**

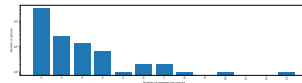

**J: 2008**

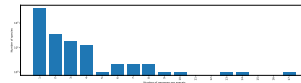

**K: 2009**

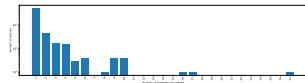

**L: 2010**

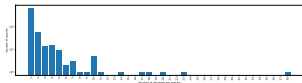

M: 2011

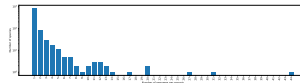

**N: 2012**

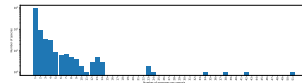

**O: 2013**

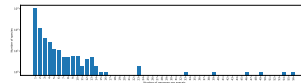

**P: 2014**

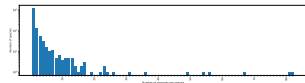

**Q: 2015**

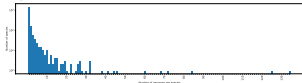

**R: 2016**

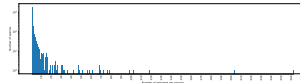

**S: 2017**

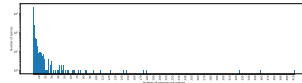

**T: 2018**

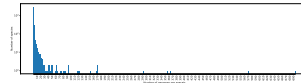

U: 2019

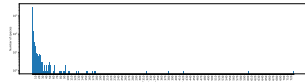

Supplement: Supplementary file 2 — Additional file 2 Histogram of the number of genomes per species. We plot the histogram of the number of genomes per species every year. The figures show that many species have only one genome. We designed our experiment so that we can train a model on some genomes of a species and then evaluate the model with other genomes of the same species. Therefore, when we train on those species, we don’t have a testing genome to simulate testing reads from. And when we do simulate testing reads from those genomes, they don’t exist in the training set. To this end, we evaluate our model with two metrics: accuracy for all reads and accuracy for known reads (reads from known species). [file 12859_2020_3744_MOESM2_ESM.pdf]

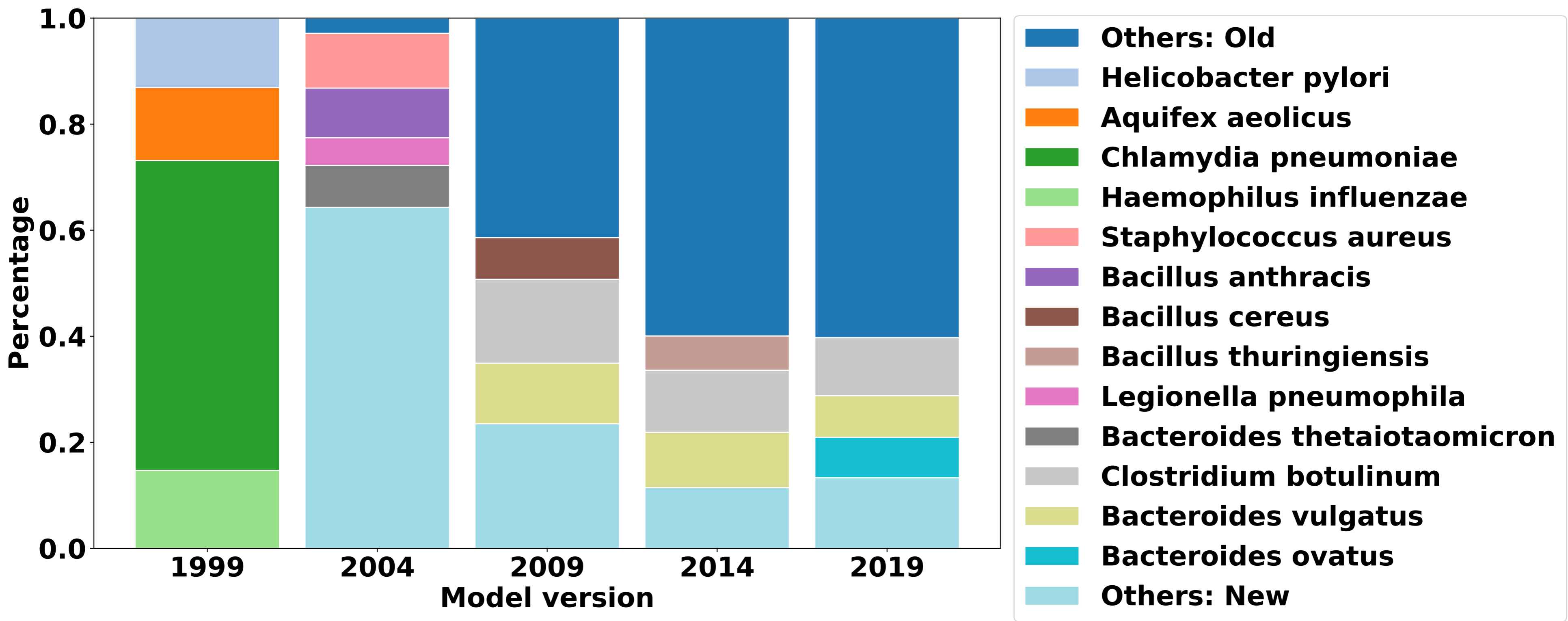

Supplement: Supplementary file 6 — Additional file 6 Profiling results change over time on species level. The NBC incremental learning classifiers trained on different years are evaluated on a real human fecal sample (SRA ID: SRS105153) on species level. The figure shows that the predicted composition of the sample changed over time and is influenced by the training data. Taxa with lower than 5% relative abundance is moved to either “Others: New” category or “Others: Old” category depending on weather the taxa is recently added or was added in previous section. [file 12859_2020_3744_MOESM6_ESM.pdf]

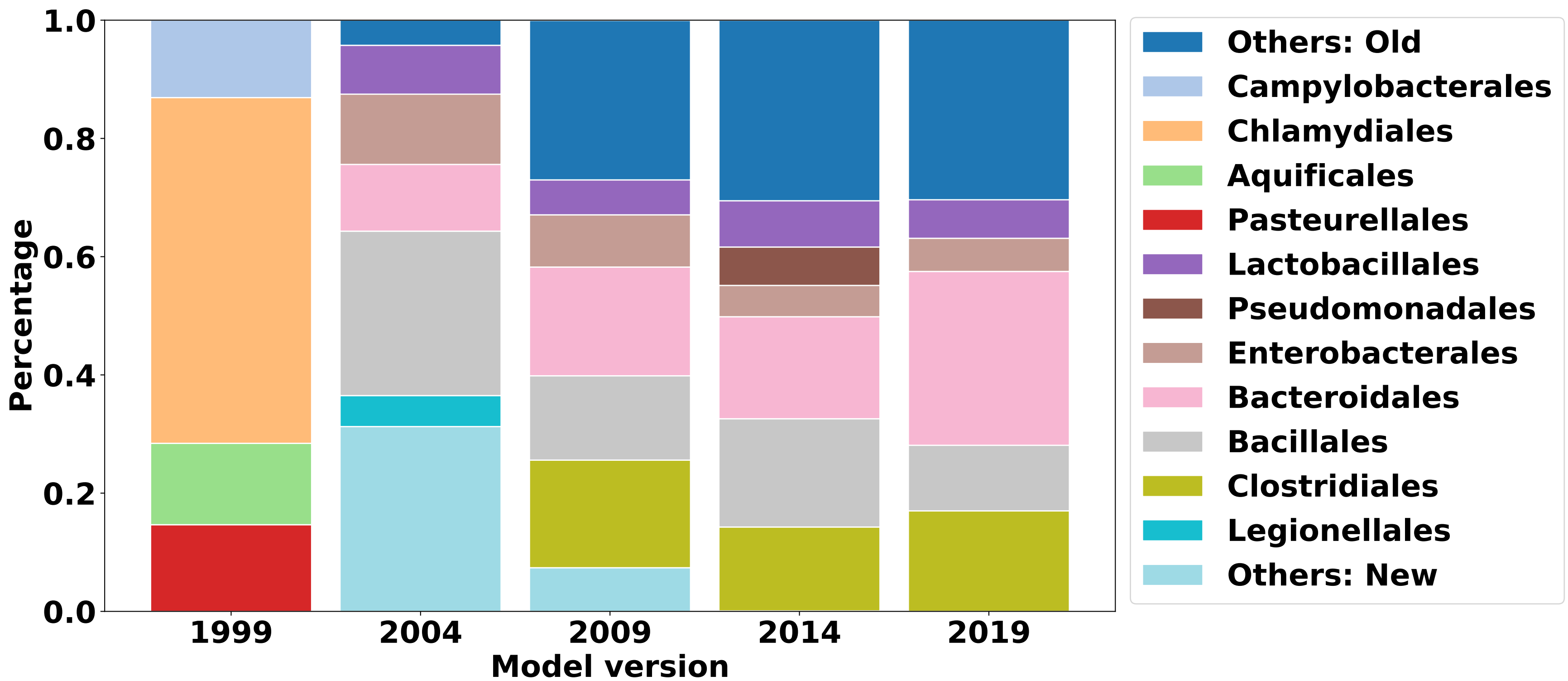

Supplement: Supplementary file 7 — Additional file 7 Profiling results change over time on order level. The NBC incremental learning classifiers trained on different year are evaluated on a real human fecal sample (SRA ID: SRS105153) on order level. The figure shows that the predicted composition of the sample changed over time and is influenced by the training data. Taxa with lower than 5% relative abundance is moved to either “Others: New” category or “Others: Old” category depending on weather the taxa is recently added or was added in previous section. [file 12859_2020_3744_MOESM7_ESM.pdf]

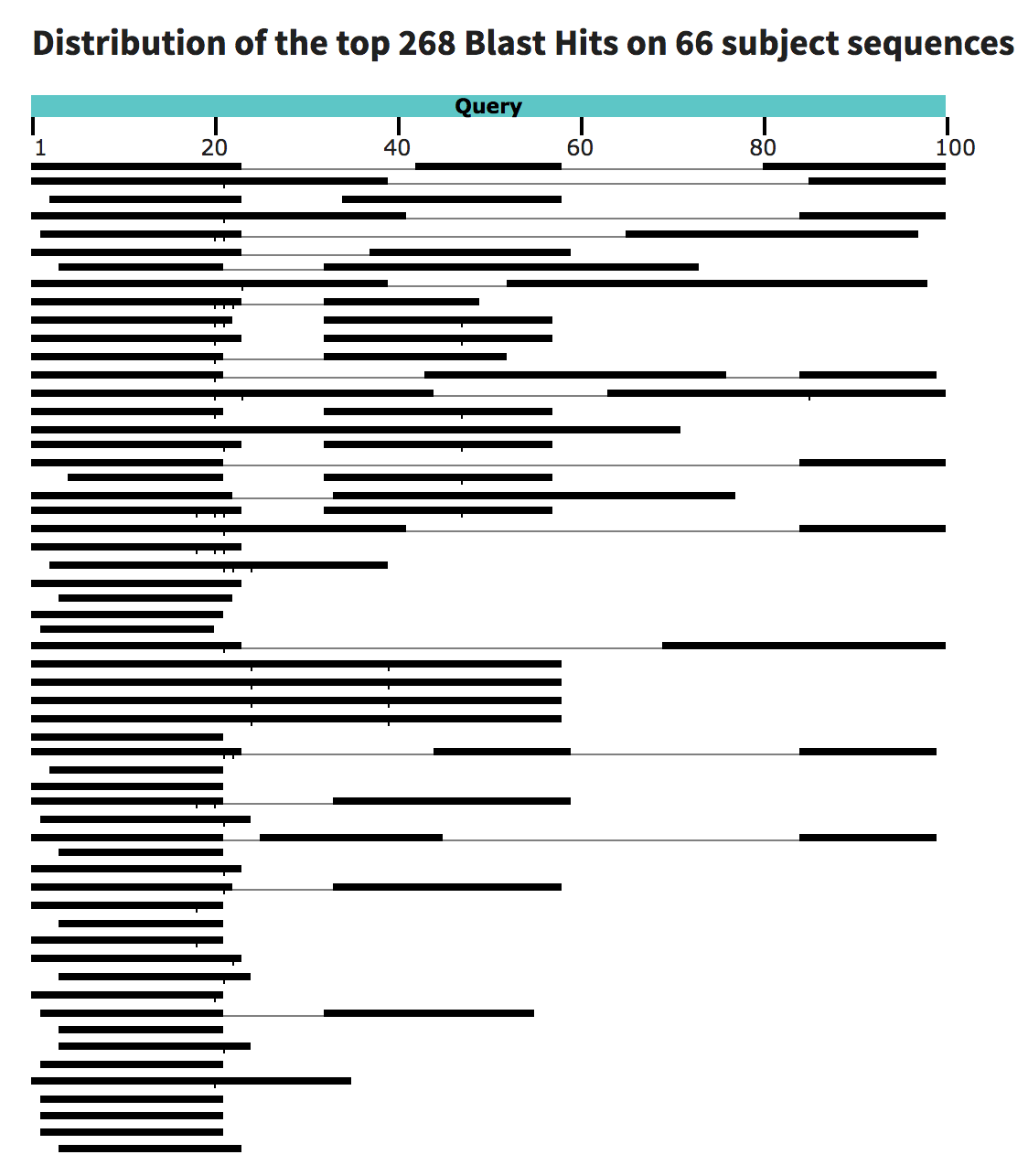

Supplement: Supplementary file 11 — Additional file 11 Blast result for B. aphidiciola (taxid: 9). Blast result for the case example read in “Case example of B. aphidicola and C. botulinum misclassification” section against B. aphidiciola (taxid: 9) exclusing strain Schlechtendalia chinensis. [file 12859_2020_3744_MOESM11_ESM.png]

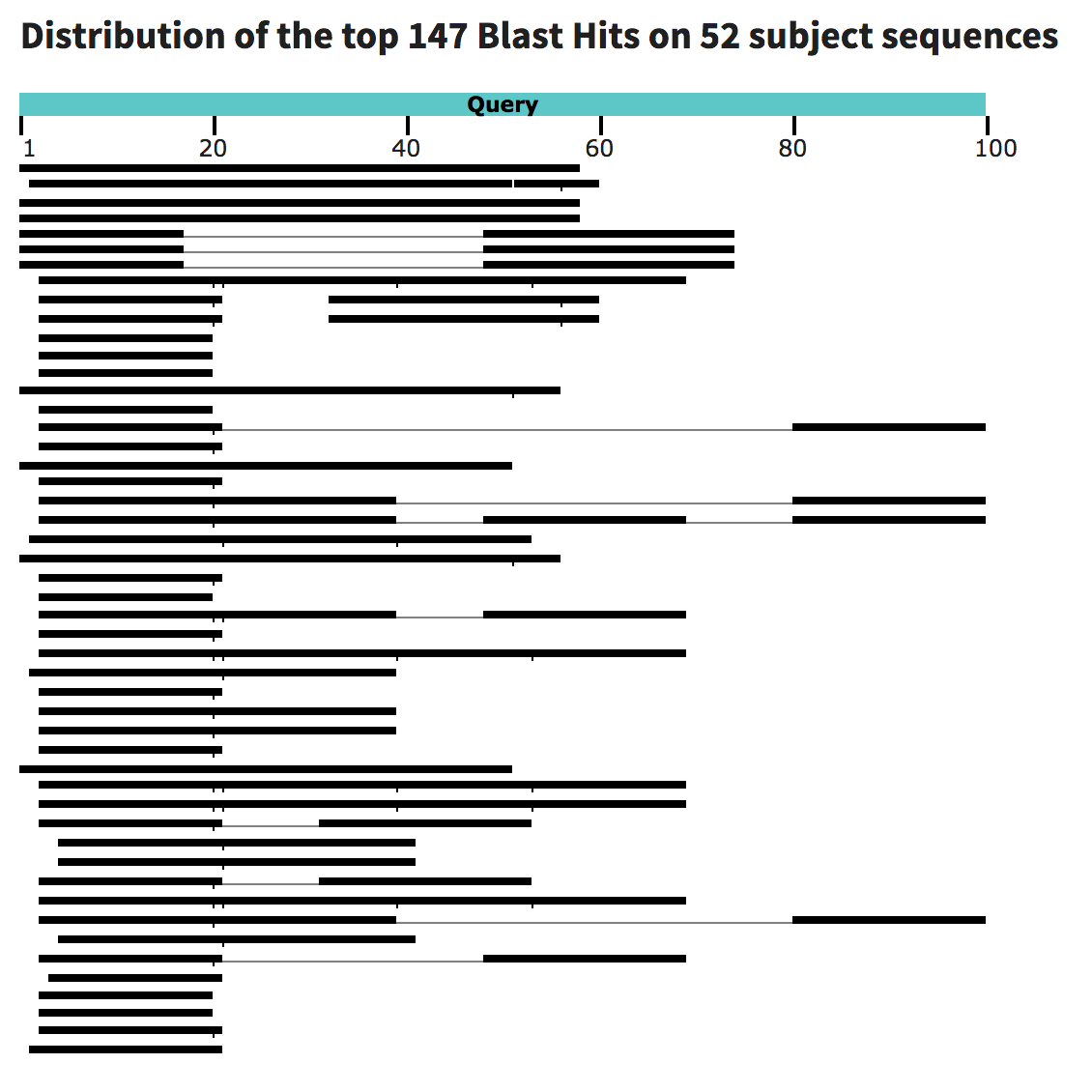

Supplement: Supplementary file 12 — Additional file 12 Blast result for C. botulinum (taxid:1491). Blast result for the case example read in “Case example of B. aphidicola and C. botulinum misclassification” section against C. botulinum (taxid:1491). [file 12859_2020_3744_MOESM12_ESM.png]
